# Thomas Edward Turner, D.D.S.

**Published:** 1915-02

**Authors:** 


THOMAS EDWARD TURNER, D.D.S.—Dr. Turner died in his 47th year, at St. Louis, November 14, 1914, as the result of an automobile accident.

Dr. Turner was born in Carrollton, Mo., June 22, 1868. He graduated from Washington University Dental School in 1890. After practicing for six years in Neosho, Mo., he moved to St. Louis and was associated with Dr. Holmes. In 1904 he was appointed a member of the Missouri State Board of Examiners, which position he retained until his death. Dr. Turner has taken great interest in the work of the National Association of Dental Examiners and did much to keep that organization intact and make its work effective He was actively interested in the National Dental Association and was one of its Vice-Presidents at the time of his death. He will be seriously missed in the councils of the dentists of Missouri and St. Louis, in all of which he was very active.

